# Study on the Mechanism of Nd:YAG Laser-Assisted Therapy on the Changes of Subgingival Flora in Periodontitis

**DOI:** 10.3290/j.ohpd.b5795663

**Published:** 2024-10-24

**Authors:** Yuhang Xie, Yi Peng, Ting Zhou, Shaowen Lu, Jianhua Wu

**Affiliations:** a Attending Physician, Department of the Second Clinic, Kunming Medical University School and Hospital of Stomatology, Kunming City, Yunnan Province, China; Yunnan Key Laboratory of Stomatology, Kunming City, Yunnan Province, China. Wrote the manuscript, analyzed the results.; b Associate Professor, Department of Periodontology, Kunming Medical University, School and Hospital of Stomatology, Kunming City, Yunnan Province, China; Yunnan Key Laboratory of Stomatology, Kunming City, Yunnan Province, China. Performed statistical evaluation.; c Associate Professor, Department of Orthodontics, Kunming Medical University School and Hospital of Stomatology, Kunming City, Yunnan Province, China; Yunnan Key Laboratory of Stomatology, Kunming City, Yunnan Province, China. Contributed substantially to discussion.; d Attending Physician, Department of Periodontology, Kunming Medical University School and Hospital of Stomatology, Kunming City, Yunnan Province, China; Yunnan Key Laboratory of Stomatology, Kunming City, Yunnan Province, China. Advisor, proofread the manuscript.; e Associate Chief Physician, Department of Periodontology, Hospital of Stomatology, Kunming City, Yunnan Province, China; Yunnan Key Laboratory of Stomatology, Kunming City, Yunnan Province, China. Contributed substantially to discussion.; * Yuhang Xie and Yi Peng as co-first author contributed equally to this paper.

**Keywords:** periodontitis, Nd:YAG laser, red complex, 16S amplicon sequencing

## Abstract

**Purpose:**

This study evaluated the therapeutic efficacy of combining Neodymium-doped Yttrium Aluminum Garnet (Nd:YAG) laser with subgingival curettage and root planing (SRP) in generalised stage III/grade C periodontitis patients and its effects on cytokine dynamics and microbial community.

**Materials and Methods:**

Fifteen patients diagnosed with stage III/grade C periodontitis were included in the cohort. The right and left sides of the mouth were randomly assigned either the conventional SRP (control) group or the SRP supplemented with Nd:YAG laser group (experimental group, 160 mJ, 4 W) in a split-mouth design. Clinical periodontal indices were recorded at baseline and at the 6-week follow-up post-treatment. ELISA was utilised to measure IL-1β and TNF-α levels in gingival crevicular fluid. The subgingival microbiota’s composition and variations were characterised using 16S rDNA amplicon sequencing, while quantitative real-time polymerase chain reaction (qRT-PCR) was employed to analyse the changes in the red-complex bacteria in subgingival plaque.

**Results:**

The SRP+Nd group exhibited a statistically significant reduction in record probing depth (PD) and bleeding on probing (BOP) compared to the SRP group after treatment (p<0.05). The SRP+Nd group showed a markedly lower IL-1β level than the SRP group (p<0.05). Furthermore, there was no statistically significant difference in the dominant subgingival microbiota composition and level of the red-complex bacteria between the two groups (p>0.05).

**Conclusion:**

The adjunctive use of Nd:YAG laser with SRP demonstrates promising short-term therapeutic benefits for patients with extensive stage III/grade C periodontitis. Both SRP as a standalone treatment and its combination with Nd:YAG laser effectively facilitate a transition in the dominant bacterial community from periodontitis-associated to periodontal health-associated microbiota.

Periodontitis is a chronic inflammatory and destructive disease caused by the colonisation of periodontal pathogens in the subgingival area, disrupting the original microecological balance, triggering the host’s immune responses, Certain pathobionts can orchestrate the establishment of dysbiotic communities that can subvert the host immune system, triggering inflammation and tissue destruction.^18^ In addition, immune pathogenesis includes host genetics, lifestyle, stress, and systemic conditions,^3^ and can ultimately lead to the destruction of periodontal tissues and alveolar bone resorption.^16^ It is currently the main reason for tooth loss in adults in China and a high-risk factor for various systemic diseases. Periodontitis is an insidious inflammatory condition, precipitated by the subgingival colonisation of periodontopathic microorganisms. This colonisation perturbs the indigenous microecological equilibrium, incites a host immune response, and culminates in the progressive degradation of periodontal tissues and the resorption of alveolar bone.^4^ Subgingival scaling and root planing (SRP) are currently effective conventional treatment methods.^19^ However, SRP treatment alone may be unpredictable at times and unsuccessful at eliminating the pathogenic bacteria owing to their location within the periodontal tissues or in other sites unreachable by periodontal instruments during debridement, such as initially deep periodontal pockets, furcation-involved lesions and root concavities.

The Neodymium-doped Yttrium Aluminum Garnet (Nd:YAG) laser, with its 1064-nm wavelength, exhibits good tissue penetration and possesses superior tissue ablation capabilities, coupled with bactericidal and detoxifying properties, all without incurring substantial thermal damage to adjacent tissues.^24^ This technology holds promise as an adjunctive modality to SRP, augmenting its therapeutic impact. Nonetheless, scholarly discourse persists within the academic community concerning the synergistic efficacy of SRP integrated with Nd:YAG laser therapy in the management of periodontitis.

The oral cavity is a reservoir for an great diversity of over 300 bacterial strains, second only to the complexity of the gastrointestinal microbiome.^31^ Socransky et al^26^ have divided subgingival bacteria into six predominant microbial complexes, elucidating their respective roles throughout the progression of periodontal inflammation. However, the scope of traditional periodontal microbial detection methods is limited, precluding a comprehensive characterisation of the microbial flora. High-throughput sequencing technologies (HTS), with their wide sequencing spectrum, are capable of concurrently analysing tens of millions of genetic sequences,^36^ offering a comprehensive appraisal of microbial community dynamics. Furthermore, these methodologies are adept at detecting low-abundance bacterial species that elude the detection capabilities of conventional techniques.^5^ Given the disparity in dominant bacterial communities between individuals with good oral health and those afflicted with periodontitis, in addition to the stratified distribution of these dominant bacteria at varying depths at the same tooth position,^27^ elucidating the dynamics of these microbial populations is instrumental for disease aetiology analysis, personalised therapeutic intervention, and prognostic evaluation. Periodontal probe diagnostic imaging can evaluate the level and degree of bone destruction and bone defect morphology.^15^ However, there is scant literature on whether discernible variations in the subgingival bacterial shifts following Nd:YAG laser-assisted SRP exist in comparison to traditional SRP.

Therefore, we propose a hypothesis that changes in oral microbiota and expression levels of inflammatory factors play an important role in evaluating the efficacy of Nd: YAG laser combined with subgingival scaling and root planing (SRP) in the treatment of patients with generalised grade III/C periodontitis. This investigation employed 16S amplicon sequencing to delineate the temporal dynamics of the subgingival microbiota in patients pre- and post-treatment. Quantitative real-time polymerase chain reaction (qRT-PCR) was utilised to assess alterations in the red complex bacteria, which comprise *Porphyromonas gingivalis* (Pg), *Treponema denticola* (Td), and *Tannerella forsythia* (Tf). Concurrently, clinical periodontal indices were measured, and enzyme-linked immunosorbent assay (ELISA) was conducted to quantify the levels of cytokines IL-1β and TNF-α. The study aimed to provide a comprehensive analysis of the short-term therapeutic mechanisms of Nd:YAG laser in conjunction with SRP for patients with advanced stage III/grade C periodontitis.

## MATERIALS AND METHODS

### Ethical Statements

This study was approved by the Medical Ethics Committee of the Affiliated Stomatological Hospital of Kunming Medical University (approval number: KYKQ2020MEC012). All participants signed an informed consent form, ensuring the study’s adherence to ethical standards.

### Research Subjects

A prospective cohort study was carefully developed using G* Power 3.1 software (Düsseldorf, Germany) for calculating the minimum number of samples required using a paired t-test with a significance level of 0.05, test efficacy of 0.8, and effect size of 0.8. A minimum sample size of 15 cases was estimated. A total of 20 patients with periodontitis were enlisted from the Department of Periodontitis at the Affiliated Stomatological Hospital of Kunming Medical University between September 2019 to December 2020 , forming the study population. Two participants were lost to follow-up during the study, and the qRT-PCR amplification of plaque samples from three additional patients was unsuccessful. As a result, the final analysis comprised 15 patients, with a mean age of 36.80 ± 11.47 years, and a range of ages from 23 to 58 years. Six males and nine females comprised the cohort.

The eligibility criteria for patient enrollment in the study were:

1. conformity with the diagnostic criteria for generalised stage III/grade C periodontitis;^6^
2. no periodontal interventions within the preceding six months;
3. no administration of antibiotics or non-steroidal anti-inflammatory medications within the last three months;
4. presence of systemic conditions that do not interfere with periodontal therapy, with female participants neither pregnant nor lactating;
5. absence of a smoking history;
6. no prior history of orthodontic intervention;
7. demonstration of good compliance with the study protocol.

The inclusion criteria for the affected teeth were:

1. A minimum of 4 affected teeth (excluding third molars) in each half of the patient’s mouth, meeting the specified conditions: a) probing depth (PD) at the deepest site ≥6 mm, clinical attachment loss (CAL) at the deepest site ≥5 mm, and radiographic evidence of alveolar bone loss amounting to 1/2 to 2/3 of the root length, with positive bleeding on probing (BOP);
2. Presence of coronal edentulous necks with full crown restorations;
3. Molar furcation involvement classified as either Class II or III. The classification proposed by Glickman divides bifurcation lesions into four classes according to the degree of involvement. Grade II involvement: the bone is destroyed on one or more aspects of the furcation, but a portion of the alveolar bone and periodontal ligament remain intact. Grade III involvement: the inter-radicular bone is completely absent, but the facial and/or lingual orifices of the furcation are occluded by gingival tissue;^6,19^
4. Absence of any root fractures, root fissures, or developmental defects in tooth tissue;
5. Tooth mobility should be rated less than Grade II.^1^

These criteria were applied to ensure that the selected teeth were representative of the periodontal conditions under investigation and to standardise the study sample.

### Experimental Design

The periodontal status of patients was documented on a periodontal chart to determine eligibility for the study after they underwent supragingival scaling surgery and received oral hygiene instruction. Based on the inclusion criteria, four teeth were selected on the left and right sides each patient’s dentition, resulting in a total of eight teeth per patient. A total of 120 teeth from all participants were identified as afflicted.

These teeth were subjected to initial clinical index documentation and sample collection at baseline. Subsequently, using a simple randomisation method, the teeth on the left and right sides in each patient were allocated to either the SRP treatment group (15 participants contributing to a total of 60 teeth) or the SRP+Nd treatment group (same 15 participants contributing to an additional 60 teeth). The study was designed as a self-controlled experiment, with participants receiving distinct treatments on opposite halves of their mouth simultaneously. One side acted as the experimental group, while the other served as the control. This approach helped to mitigate the impact of individual variability. Only the individual responsible for the randomisation and the treating clinician were aware of which side received laser treatment. Participants, those recording clinical data, and laboratory staff were blinded to the group assignments. This blinding protocol was essential to minimise bias and expectancy effects in the study.

### Periodontal Treatment

According to the grouping situation, the SRP group was treated with an ultrasound therapy instrument (Piezon Master 700, EMSM; Nyon, Switzerland) and hand instruments (Gracey Curets, Hu-Friedy; Chicago, IL, USA) (periodontal curettes 5/6, 7/8, 11/12, 13/14). On the day of SRP treatment and two weeks later, teeth in the SRP+Nd group were treated with the laser, consisting of periodontal pocket irradiation using a pulsed Nd: YAG laser (Nd:YAG Laser Therapy Device, Sichuan Aerospace World Technology; Sichuan, China) with a wavelength of 1064 nm (160 mJ, 25 Hz, 4 W, water level 2, air level 3),^28^ with a fiber- optic probe diameter of 600 μm. The fiber was carefully inserted into the base of the periodontal pocket, aligning it parallel to the root surface. As the laser light was emitted, the fiber was manipulated to perform a slow “Z” motion, lifting and pulling from the apical to the coronal aspect of each tooth surface. This motion was executed in a sweeping manner, covering the mesial to distal and buccal to lingual aspects of the teeth. Depending on the area of the periodontal pocket, the irradiation time for a single tooth was 40 s, and for a multi-rooted tooth, it was 60 s, with an average power density of 1415 W/cm^2^.

### Evaluation of Periodontal Clinical Indicators

The clinical indicators, including probing depth (PD), clinical attachment loss (CAL), bleeding on probing (BOP), and plaque Index (PLI), were evaluated. These measurements were taken at baseline and again at the 6-week post-treatment interval.^10^

### Evaluation of Cytokines

At baseline, the deepest PD site from each of the six sites of the included teeth was selected for the collection of gingival crevicular fluid (GCF). For the side treated with SRP, the fluid samples from four sites of four teeth were combined into a single sample. Likewise, for the side treated with SRP+Nd, the GCF samples from the four sites of four teeth were also combined into a single sample, each then stored in individual tubes. Six weeks post-treatment, samples were collected again from the same sites as initially sampled. A total of 60 GCF samples were collected for the study. The rationale for this method was to ensure consistency with the subgingival plaque collection sites, thereby simplifying the subsequent statistical analysis.

Prior to GCF collection, participants were instructed to rinse their mouths with water, after which the area was isolated using cotton rolls and the tooth surface was dried. Filter paper was then inserted into the periodontal pocket until a slight resistance was encountered, left in place for 30 s, and subsequently removed. The filter paper was transferred into an Eppendorf tube, to which 750 μl of phosphate buffered saline (PBS) solution was added. The samples were then stored at -20°C for subsequent analysis.

The concentrations of IL-1β and TNF-α in the GCF samples were determined using a sandwich ELISA method. The assay was performed strictly in accordance with the protocol outlined in the kit instructions (Human IL-1β/TNF-α ELISA Kit, Proteintech; Wuhan, China).

### 16S Amplicon Sequencing

At baseline, subgingival plaque was collected from the site with the deepest PD on each tooth among the six sites of the included teeth, aligning with the method used for GCF collection. The plaque from four teeth on the side designated for conventional SRP was pooled into one sample, and the plaque from the four teeth on the SRP+Nd treatment side was similarly pooled, with each sample stored in separate tubes. This process was repeated at the 6-week post-treatment follow-up, resulting in a total of 60 subgingival plaque samples for the study.

Subgingival plaque was collected using Gracey curettes immediately after GCF collection, both at baseline and at the 6-week follow-up, targeting the same sites each time. The samples were then stored at -80°C for future analysis. This approach ensured consistency with the GCF collection sites and allowed for a comprehensive comparison of data before and after treatment.

Genomic DNA was extracted from subgingival plaque samples using a DNA extraction kit (MP Biomedicals; Santa Ana, CA, USA). The purity and concentration of the DNA were assessed using a NanoDrop 2000 spectrophotometer (Thermo Fisher Scientific; Waltham, MA, USA). PCR amplification targeted the V3-V4 region of the 16S rRNA gene, utilising the upstream primer 338F (ACTCCTACGGGGAGGCAGCAG) and the downstream primer 806R (GGACTACHVGGGTWTCTAAT). After quality assessment, PCR-purified products underwent 16S rDNA amplification. A sequencing library was constructed using the NEXTFLEX Rapid DNA-Seq Kit (5144-08, Bioo Scientific; Austin, TX, USA), and sequencing was conducted on Illumina’s Miseq PE300 platform.^35^

The QIIME2 plugin was utilised to process the raw sequencing data into a feature sequence table. Operational Taxonomic Units (OTUs) were picked from the optimised sequences, clustered based on 99% similarity against the GREENGENES database,^32^ with a 70% similarity threshold applied to derive the taxonomic classification table. The core diversity plugin in QIIME2 was employed to calculate the diversity matrix and the Alpha (Shannon) diversity index at the feature sequence level, and to ascertain the relative abundance of each taxonomic category and species. The Linear Discriminant Analysis Effect Size (LEfSe) was applied to determine if there were significant differences in the taxonomic composition of the subgingival microbiota among the groups.^8^

### qRT-PCR

Total RNA was extracted from plaque samples using Trizol lysate (catalog number 15596026, Thermo Fisher Scientific / Life Technologies; Carlsbad, CA,USA). The RNA was then reverse transcribed into complementary DNA (cDNA) with the FastKing RT kit (KR116, Tianjin, China). The cDNA was subsequently added to the components of the SYBR Premium EX Taq kit (RR420A; Takara, Japan) for qRT-PCR analysis. The qRT-PCR was conducted on real-time fluorescence qPCR instruments (ABI7500, Applied Biosystems; Foster City, CA, USA), with triplicate wells for each sample to ensure accuracy. Primers for real-time fluorescence quantitative PCR were designed based on the 16S rRNA gene sequences of Pg, Td, and Tf obtained from GenBank in the National Center for Biotechnology Information (NCBI). All primers were custom synthesised by Sangon Biotech (Shanghai, China), as detailed in Table 1.

**Table 1:** Gene primer sequence information

Primer name		Sequence (5´to 3´)
*Tannerella forsythia (Tf)*	Forward Primer	CGCAGAAGGTGAAAGTCCTGTAT
	Reverse Primer	TGTGACGGGCGGTGTGTA
*Porphyromonas gingivalis (Pg)*	Forward Primer	ATCCTGGCTCAGGATGAACG
	Reverse Primer	TACGCATACCCATCCGCAA
*Treponema denticola (Td)*	Forward Primer	AGAGCAAGCTCTCCCTTACCGT
	Reverse Primer	TGCACCATTCAACTCCTCGC


The gene copy numbers of Pg, Td, and Tf in the subgingival plaque samples were quantified using standard curves derived from qRT-PCR, followed by logarithmic transformation to normalise the data.

### Statistic Analysis

All data were analysed using SPSS 21.0 (IBM; Armonk, NY, USA). The measurement data are represented as mean ± SD. Data data between the two groups were analysed using independent-sample t-tests. The data analysis between multiple groups was conducted using one-way ANOVA, followed by Tukey’s post-hoc test.

## RESULTS

### Clinical Indicator Test Results

Changes in PD, CAL, BOP, and PLI before and after treatment were examined, and the results showed a statistically significant reduction in all of these indicators after treatment (p<0.05). Notably, the SRP+Nd group exhibited a more pronounced reduction in PD and BOP compared to the SRP group (p<0.05), with a particularly statistically significant decrease in the number of sites with PD ≥ 6 mm in the SRP+Nd group (p<0.05). However, no statistically significant differences were detected between the two groups in terms of changes in CAL and PLI (p>0.05) (Table 2).

**Table 2:** Detection results of PD, AL, BOP, and PLI (mean±SD) (n=15)

Measurement value	Timepoint	SRP	SRP+ Nd
PD (mm)	Baseline	4.84±0.61	4.79±0.56
	After treatment	3.12±0.59*	2.85±0.50*#
PD ≥6 mm Loci (number)	Baseline	8.27±3.12	8.20±3.08
	After treatment	2.20±1.82*	1.27±1.16*#
AL (mm)	Baseline	4.96±0.72	4.82±0.69
	After treatment	3.48±0.86*	3.28±0.73*
BOP (%)	Baseline	78.60±14.58	83.33±12. 60
	After treatment	32.78±19.21*	8.88±11.12*#
PLI	Baseline	3.59±0.52	3.71±0.46
	After treatment	2.87±0.55*	2.79±0.59*

*Statistically significant difference between the group and the baseline, p<0.05; # Statistically significant difference between the two groups at the same time point, p<0.05.

### Cytokine Levels

There was no statistically significant difference in the levels of IL-1β and TNF-α between the two groups at baseline (p>0.05), but both decreased statistically significantly after treatment compared to baseline (p<0.05). Intergroup comparison showed that the IL-1β level in the SRP+Nd group was statistically significantly lower than that in the SRP group after treatment (p<0.05), and there was no statistically significant difference in TNF-α levels between the SRP group and the SRP+Nd group after treatment (p>0.05) (Table 3).

**Table 3:** IL-1β and TNF-α content (mean ± SD) (n=15)

Measurement value	Timepoint	SRP	SRP+ Nd
IL-1β (pg/ul)	baseline	14.10±2.59	14.14±3.37
	After treatment	4.07±0.98*	3.08±1.27*#
TNF-α (pg/ul)	baseline	8.28±2.21	8.36±1.67
	After treatment	2.30±0.45*	2.14±0.42*

*Statistically significant difference between the group and the baseline, p<0.05; # Statistically significant differencebetween the two groups at the same time point, p<0.05.

### 16S Amplicon Sequencing

#### Species composition analysis

A total of 60 subgingival plaque samples were collected in this experiment, and a total of 2240595 high-quality sequences were obtained. A total of 1746 Operational Taxonomic Units (OTUs) were detected, classified into 1 kingdom, 17 phyla, 33 classes, 55 orders, 92 families, 147 genera, and 190 species.

**Fig 1:**
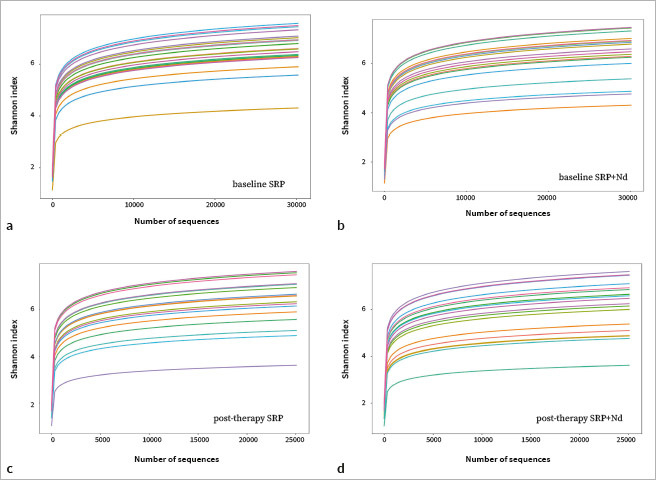
α diversity dilution curve.

In α diversity analysis, the dilution curves of the SRP baseline group, SRP+Nd baseline group, SRP post-treatment group, and SRP+Nd post-treatment group all tended to flatten out, indicating that the sequencing results were sufficient to reflect the diversity included in the current sample (Fig 1). Intragroup comparison showed that compared to baseline, the Shannon index of both the SRP group and the SRP+Nd group showed a decreasing trend after treatment, but the difference was not statistically significant (p>0.05). The comparison between groups showed that there was no statistically significant difference in Shannon index between the SRP group and the SRP+Nd group after treatment (p>0.05) (Table 4).

**Table 4:** α diversity index comparison (mean ± SD) (n=15)

Shannon index	Baseline	After treatment
SRP	6.15±0.75	5.89±0.76
SRP+ Nd	6.24±0.55	5.83±1.02


#### Analysis of relative abundance of subgingival microbiota

**Fig 2:**
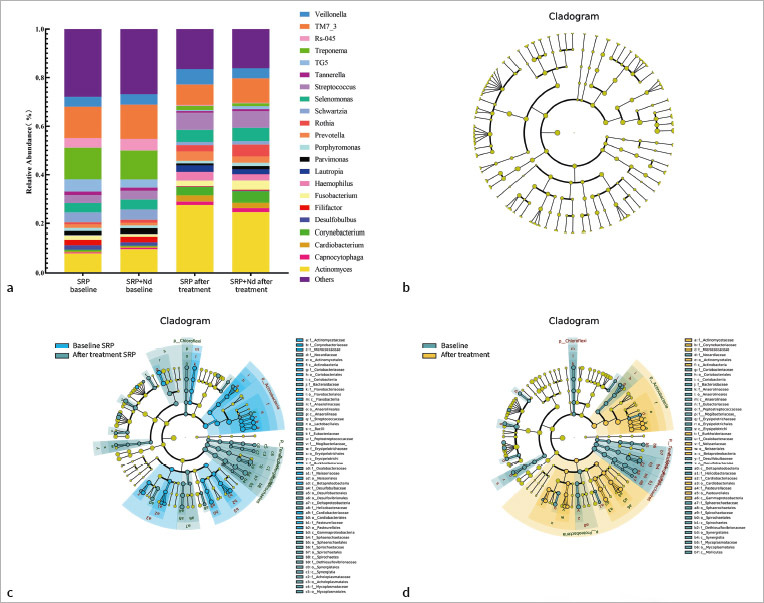
16S amplicon sequencing analysis of bacterial abundance. A: Bar chart of relative species abundance; B: Cladogram diagram of LEfSe analysis; C: LEfSe analysis of the SRP group using a cladogram plot; D: LEfSe analysis cladogram of SRP+Nd group.

A total of 147 bacterial genera were detected, of which 22 genera had a relative abundance greater than 1% (Fig 2). At baseline, there was no statistically significant difference in the composition of subgingival microbiota between the SRP group and the SRP+Nd group (p>0.05). There was also no statistically significant difference in the composition of subgingival microbiota between the two groups after treatment (p>0.05). Comparing before and after treatment, the relative abundance of 6 genera of bacteria, including *Treponema*, decreased statistically significantly (p<0.05), while the relative abundance of 6 genera of bacteria, including *Actinomyces*, increased statistically significantly (p<0.05). The relative abundance of 10 genera of bacteria, including Capnocytophaga, did not show statistically significant changes (p>0.05) (Table 5).

**Table 5:** Relative abundance changes of subgingival microbiota levels (n=15)

Genus	SRP		SRP+Nd	
	Baseline	After treatment	Baseline	After treatment
*Actinomyces*	7.99	27.54*	9.81	24.63*
*Capnocytophaga*	0.42	1.46	0.48	1.61
*Cardiobacterium*	0.39	2.49*	0.48	2.21*
*Corynebacterium*	0.79	3.53*	0.47	4.78*
*Desulfobulbus*	1.78	0.11*	1.43	0.23
*Filifactor*	2.19	0.25*	2.24	0.36*
*Fusobacterium*	1.91	2.21	1.08	3.85*
*Haemophilus*	0.02	3.62*	0.02	2.55*
*Lautropia*	0.07	2.64*	0.07	2.04*
*Parvimonas*	1.33	0.87	1.96	1.34
*Porphyromonas*	1.18	0.97	1.11	1.35
*Prevotella*	1.49	3.93	1.06	2.63
*Rothia*	0.82	2.54*	1.23	4.81*
*Schwartzia*	4.06	1.34*	4.28	1.49*
*Selenomonas*	3.87	4.99	4.06	5.45
*Streptococcus*	3.32	7.08	3.61	6.95
*Tannerella*	1.42	0.66*	1.38	0.81*
*TG5*	5.04	0.44*	3.34	1.13*
*Treponema*	13.07	1.66*	11.91	1.25*
TM7-3	3.89	0.44	4.68	0.26
Rs-045	12.88	8.38*	14.17	9.93*
*Veillonella*	4.19	6.32	4.38	4.18

*Post-treatment compared with pre-treatment, p<0.05.

LEfSe analysis can more intuitively display bacterial genera abundance with statistically significant differences between the two groups, as shown in Fig 2. There were statistically significant differences in bacterial genera abundance before and after treatment within the group (p<0.05), while there was no statistically significant difference in bacterial genera between the SRP and SRP+Nd groups after treatment (p>0.05) (Fig 2).

### Red Complex Detection

Compared with baseline, the number of Pg statistically significantly decreased (p<0.05) in both the SRP group and the SRP+Nd group after treatment, while the number of Tf and Td decreased only slightly (p>0.05). A comparison between groups showed that there was no statistically significant difference in the number of Pg, Tf, and Td between the SRP group and the SRP+Nd group after treatment (p>0.05) (Table 6).

**Table 6:** Number detection of red complex Pg, Tf, and Td (mean ± SD) (n=15)

	Group	Baseline	After treatment
Pg [lg(copy/μl)]	SRP	4.39±1.20	2.97±0.79*
	SRP+ Nd	4.70±0.93	3.29±0.63*
Tf [lg(copy/μl)]	SRP	4.37±1.65	4.04±1.36
	SRP+ Nd	4.79±0.77	4.64±1.10
Td [lg(copy/μl)]	SRP	4.02±1.31	3.60±1.19
	SRP+ Nd	4.26±0.78	4.11±0.80

*Post-treatment compared with pre-treatment, p<0.05.

## DISCUSSION

Risk factors are an evolving area that will continue to change as the understanding of periodontitis diseases increases.^9^ Patients with extensive stage III/grade C periodontitis exhibit severe periodontal tissue lesions, increased alveolar bone resorption, and the potential for root bifurcation lesions and tooth loss. Consequently, the prognosis is highly ambiguous and the treatment for this type of patient is highly complex. This study employed a split trial design to evaluate the short-term efficacy of Nd:YAG laser combined with SRP in patients with extensive stage III/grade C periodontitis, and to analyse the changes in clinical periodontal indices, cytokines, subgingival microbiota and number of red-complex bacteria before and after treatment.

The periodontal clinical indices showed that compared with the pre-treatment baseline, both the SRP-treated group and the SRP-plus-Nd:YAG-treated group demonstrated statistically significant reductions in subjects’ PD, CAL, BOP, and PLI. Further findings indicated that compared to the SRP-treated group, the SRP-plus-Nd:YAG-treatment further down-regulated the levels of PD and BOP, suggesting that the SRP-combined Nd:YAG-treatment is more effective in reducing PD and BOP than the SRP-treated approach.

This result suggests that SRP provides the primary solution to periodontitis and that Nd:YAG can be used as an adjunct to SRP in the treatment of periodontitis to improve periodontal status. Research has indicated that Nd:YAG laser favours wound healing and inhibits inflammation, for instance, by inducing gingival fibroblasts and periodontal ligament proliferating cells, stimulating the expression of epidermal growth factor, collagen type I, platelet-derived growth factor and basic fibroblast growth factor, and inhibiting the expression of TNF-α by bone marrow mesenchymal stem cells in an inflammatory milieu.^11^ These mechanisms may account for the reduction in PD and BOP after combination therapy. After treatment, there was no statistically significant difference in CAL between the SRP group and the SRP+Nd group, which is consistent with the meta-analysis results on Nd:YAG laser combined with non-surgical periodontal treatment.^29^ However, the levels of CAL and PLI were not statistically significantly different between the SRP-treated and SRP-plus-Nd: YAG-treated groups, the reason for which may be related to the duration and frequency of laser application and the severity of periodontal disease. The treatment parameters of the Nd:YAG laser in this study were 4 W, 160 mJ, 25 Hz, which are effective parameters for the treatment of periodontitis in our clinic. In another study, the treatment parameters of Nd:YAG laser were 0.5W, 10 Hz and 2 W, 200 mJ, 10 Hz, both of which statistically significantly reduced PD, CAL, and BOP.^19,23^ Miyazaki et al^20^ also reported that Nd:YAG laser (2 W, 100 mJ) treatment for 4 weeks statistically significantly improved CAL parameters. Zhang et al^37^ demonstrated a statistically significant improvement in PD and CAL values after 3 months of Nd:YAG treatment compared to pre-treatment. Thus, different laser energy parameters may lead to different results.

Suppression of the inflammatory response is one of the guidelines for the treatment of periodontitis; IL-1β and TNF-α are two pro-inflammatory cytokines active in periodontitis. The study showed a highly positive correlation between IL-1β levels in patients with periodontitis and clinical indices such as PD, BOP, gingival recession, and dental plaque.^33^ In the present study, the decrease in IL-1β was more pronounced after 6 weeks of treatment with SRP combined with Nd: YAG, and the combination treatment statisically significantly reduced the levels of PD and BOP, confirming the idea that IL-1β is positively correlated with PD and BOP. However, there was no statistically significant difference in TNF-α levels between the SRP+Nd group and the SRP group, which was different from the experimental results of Gómez et al^12^ and Abduljabbar et al.^2^ We hypothesise that laser energy, disease severity, and treatment time may account for the differences in experimental results.

Oral microorganisms and susceptible hosts are considered prerequisites for the development of periodontitis.^17^ Research has shown that a variety of microorganisms are associated with periodontitis, for instance, the phyla Synergistetes^34^ and Actinobacteria, and the genera* Rochella, Veyrococcus, Haemophilus, Corynebacterium*, and *Streptococcus*.^30^ At the same time, the phylum Actinobacteria, as well as the genera *Desulfobulbus, Prevotella* and *Luncomonas* were once underestimated as being associated with the progression of periodontitis, although their pathogenicity is comparable to or even higher than that of *Porphyromonas gingivalis, Forsythia*, and *Treponema gingivalis*.^13^ In this study, the relative abundance of *Tannerella, Treponema,* Actinobacteria, *Haemophilus*, and *Corynebacterium* decreased at baseline compared to 6 weeks after treatment, while the relative abundance of Actinobacteria, *Rochella, Haemophilus*, and *Corynebacterium* statistically significantly increased. This indicates that the relative abundance of pathogenic bacteria in the subgingival microbiota after SRP and SRP+Nd treatment decreased, while the relative abundance of periodontal-health–related bacteria increased. It is crucial for disease prevention and treatment to understand the role of the oral microbiome and the mechanisms of ecosystem regulation in health.^25^ Once again, it has been proven that SRP can destroy plaque biofilm, reduce the bacterial community associated with periodontitis, and increase the bacterial community associated with periodontal health, establishing a new ecological balance. It was shown that Nd:YAG laser irradiation alone did not have a statistically significant effect on any bacterial species. However, other authors have shown the combination of H_2_O_2_ and Nd:YAG to reduce the number of *Streptococcus pyogenes*, and statistically significantly reduce bacterial viability by treatment with 0.5% NaOCl and Nd:YAG.^14^ This result suggests that combination therapy with SRP + NaOCl + Nd:YAG may have an impact on periodontitis microorganisms and is a potential treatment option.

This study analysed the changes in the content of red-complex bacteria using qRT-PCR, finding no statistically significant difference in Pg, Tf, and Td copy numbers between the two groups after treatment. However, in patients with severe periodontitis, the mean level of total culturable red/orange complex periodontal pathogens per patient decreased statistically significantly from 12.0% before treatment to 4.9% after Nd:YAG laser monotherapy (59.2% decrease).^21^ Although there was no statistically significant difference in the relative abundance of subgingival microbiota between the two groups after treatment, the clinical indicators and cytokines in the SRP+Nd group statistically significantly improved. Martelli et al^22^ used Nd:YAG laser to irradiate periodontal pockets after SRP treatment. The HTS method was used to detect subgingival plaque, and the ratio of bacterial count of red, orange, and green complexes to total bacterial count was analysed. The results showed that the proportion of red and green complexes statistically significantly decreased, while the proportion of orange complexes increased. However, this experiment only measured the changes in bacterial count in red complexes before and after treatment, which has certain limitations.

## CONCLUSION

The combination of Nd:YAG laser and SRP can effectively control inflammation and promote the transition of periodontitis from active to inactive periodontitis in patients with extensive stage III/grade C periodontitis. Its short-term clinical efficacy is superior to that of SRP alone. After treatment, the subgingival dominant microbiota of patients changes from pathogenic bacteria to healthy microbiota. The long-term effects of combined Nd:YAG laser and SRP treatment on periodontitis remain to be examined in a follow-up study.

## References

[ref1] Aminoshariae A, Mackey SA, Palomo L, Kulild JC (2020). Declassifying mobility classification. J Endodont.

[ref2] Abduljabbar T, Vohra F, Kellesarian SV, Javed F (2017). Efficacy of scaling and root planning with and without adjunct Nd:YAG laser therapy on clinical periodontal parameters and gingival crevicular fluid interleukin 1-beta and tumor necrosis factor-alpha levels among patients with periodontal disease: A prospective randomized split-mouth clinical study. J Photochem Photobiol B Biol.

[ref3] Belibasakis GN, Belstrøm D, Eick S, Gursoy UK, Johansson A, Könönen E (2023). Periodontal microbiology and microbial etiology of periodontal diseases: Historical concepts and contemporary perspectives. Periodontol 2000.

[ref4] Cao Y, Qiao M, Tian Z, Yu Y, Xu B, Lao W (2018). Comparative analyses of subgingival microbiome in chronic periodontitis patients with and without IgA nephropathy by high throughput 16S rRNA sequencing. Cell Physiol Biochem.

[ref5] Chen T, Shi Y, Wang X, Wang X, Meng F, Yang S (2017). High-throughput sequencing analyses of oral microbial diversity in healthy people and patients with dental caries and periodontal disease. Molecular Medicine Reports.

[ref6] Caton JG, Armitage G, Berglundh T, Chapple ILC, Jepsen S, Kornman KS (2018). A new classification scheme for periodontal and peri-implant diseases and conditions – Introduction and key changes from the 1999 classification. J Clin Periodontol.

[ref7] Carranza FA (1979). Glickman’s clinical periodontology: prevention, diagnosis and treatment of periodontal disease in the practice of general dentistry.

[ref8] Corrêa JD, Calderaro DC, Ferreira GA, Mendonça SMS, Fernandes GR, Xiao E (2017). Subgingival microbiota dysbiosis in systemic lupus erythematosus: association with periodontal status. Microbiome.

[ref9] Darby I (2022). Risk factors for periodontitis & peri-implantitis. Periodontol 2000.

[ref10] El Mobadder M, Nammour S, Namour M, Namour A, Grzech-Leśniak K (2022). Disinfection Potential of 980 nm Diode Laser and Hydrogen Peroxide (3%) in “Critical Probing Depths” Periodontal Pockets: Retrospective Study. Life (Basel).

[ref11] Ezber A, Taşdemir İ, Yılmaz HE, Narin F, Sağlam M (2023). Different application procedures of Nd:YAG laser as an adjunct to scaling and root planning in smokers with stage III grade C periodontitis: a single-blind, randomized controlled trial. Irish J Med Sci.

[ref12] Gómez C, Domínguez A, García-Kass AI, García-Nuñez JA (2011). Adjunctive Nd:YAG laser application in chronic periodontitis: clinical, immunological, and microbiological aspects. Lasers Med Sci.

[ref13] Griffen AL, Beall CJ, Campbell JH, Firestone ND, Kumar PS, Yang ZK (2012). Distinct and complex bacterial profiles in human periodontitis and health revealed by 16S pyrosequencing. ISME.

[ref14] Golob Deeb J, Reddy N, Kitten T, Carrico CK, Grzech-Leśniak K (2023). Viability of bacteria associated with root caries after Nd:YAG laser application in combination with various antimicrobial agents: An in vitro study. Dent Med Probl.

[ref15] Heitz-Mayfield LJA (2024). Conventional diagnostic criteria for periodontal diseases (plaque-induced gingivitis and periodontitis). Periodontol 2000.

[ref16] Kwon T, Lamster IB, Levin L (2021). Current concepts in the management of periodontitis. Int Dent J.

[ref17] Luo Y, Peng X, Duan D, Liu C, Xu X, Zhou X (2018). Epigenetic Regulations in the Pathogenesis of Periodontitis. Curr Stem Cell Res Ther.

[ref18] Manoil D, Parga A, Bostanci N, Belibasakis GN (2024). Microbial diagnostics in periodontal diseases. Periodontol 2000.

[ref19] Morales A, Contador R, Bravo J, Carvajal P, Silva N, Strauss FJ (2021). Clinical effects of probiotic or azithromycin as an adjunct to scaling and root planning in the treatment of stage III periodontitis: a pilot randomized controlled clinical trial. BMC Oral Health.

[ref20] Miyazaki A, Yamaguchi T, Nishikata J, Okuda K, Suda S, Orima K (2003). Effects of Nd:YAG and CO2 laser treatment and ultrasonic scaling on periodontal pockets of chronic periodontitis patients. J Periodontol.

[ref21] McCawley TK, McCawley MN, Rams TE (2022). Immediate effect of Nd:YAG laser monotherapy on subgingival periodontal pathogens: a pilot clinical study. J Periodont Implant Sci.

[ref22] Martelli FS, Fanti E, Rosati C, Martelli M, Bacci G, Martelli ML (2016). Long-term efficacy of microbiology-driven periodontal laser-assisted therapy. Eur J Clin Microbiol Infect Dis.

[ref23] Pilloni A, Rojas MA (2018). Furcation involvement classification: a comprehensive review and a new system proposal. Dent J.

[ref24] Reynolds M, Aichelmann-Reidy M, Rosen PJETiP Lasers in Periodontal and Peri-implant Therapy: Challenges and Opportunities. Emerg Ther Periodont.

[ref25] Sedghi L, DiMassa V, Harrington A, Lynch SV, Kapila YL (2021). The oral microbiome: Role of key organisms and complex networks in oral health and disease. Periodontol 2000.

[ref26] Socransky SS, Haffajee AD, Cugini MA, Smith C, Kent RL (1998). Microbial complexes in subgingival plaque. J Clin Periodontol.

[ref27] Shi M, Wei Y, Hu W, Nie Y, Wu X, Lu RJFic (2018). The subgingival microbiome of periodontal pockets with different probing depths in chronic and aggressive periodontitis: a pilot study. Front Cell Infect Microbiol.

[ref28] Spencer P, Cobb CM, McCollum MH, Wieliczka DM (1996). The effects of CO2 laser and Nd:YAG with and without water/air surface cooling on tooth root structure: correlation between FTIR spectroscopy and histology. J Periodont Res.

[ref29] Sgolastra F, Severino M, Petrucci A, Gatto R, Monaco A (2014). Nd:YAG laser as an adjunctive treatment to nonsurgical periodontal therapy: a meta-analysis. Lasers Med Sci.

[ref30] Szafranski SP, Wos-Oxley ML, Vilchez-Vargas R, Jáuregui R, Plumeier I, Klawonn F (2015). High-resolution taxonomic profiling of the subgingival microbiome for biomarker discovery and periodontitis diagnosis. Appl Environ Microbiol.

[ref31] Teles F, Wang Y, Hajishengallis G, Hasturk H, Marchesan JT (2021). Impact of systemic factors in shaping the periodontal microbiome. Periodontology 2000.

[ref32] Turesky S, Gilmore ND, Glickman I (1970). Reduced plaque formation by the chloromethyl analogue of vitamine C. J Periodontol.

[ref33] Teles R, Sakellari D, Teles F, Konstantinidis A, Kent R, Socransky S (2010). Relationships among gingival crevicular fluid biomarkers, clinical parameters of periodontal disease, and the subgingival microbiota. J Periodontol.

[ref34] Vartoukian SR, Palmer RM, Wade WG (2009). Diversity and morphology of members of the phylum “synergistetes” in periodontal health and disease. Appl Environ Microbiol.

[ref35] Wang C, Wei S, Chen N, Xiang Y, Wang Y, Jin M (2022). Characteristics of gut microbiota in pigs with different breeds, growth periods and genders. Microb Biotechnol.

[ref36] Yang X, Que G (2020). Advance in study on 16S rRNA gene sequencing technology in oral microbial diversity. Zhong nan da xue xue bao Yi xue ban =. J Central South University Med Sci.

[ref37] Zhang Y, Tang P, Yang Q, Li C, Li L, Han M (2024). Efficacy of scaling and root planing with and without adjunct Nd:YAG laser therapy on glucose control and periodontal microecological imbalance in periodontitis patients with type 2 diabetes mellitus: a randomized controlled trial. Clin Oral Investig.

